# Non-Contact Laser Therapy for Glaucoma: A Review of Direct Selective Laser Trabeculoplasty

**DOI:** 10.3390/jcm14196884

**Published:** 2025-09-28

**Authors:** Anna M. Koziorowska, Aleksandra Opala, Iwona Grabska-Liberek

**Affiliations:** Department of Ophthalmology, The Centre of Postgraduate Medical Education, 01-416 Warsaw, Poland; anna.monika.koziorowska@gmail.com (A.M.K.);

**Keywords:** direct selective laser trabeculoplasty, transscleral trabeculoplasty, glaucoma, open-angle glaucoma

## Abstract

Glaucoma is a chronic, progressive optic neuropathy characterized by the degeneration of retinal ganglion cells. It is one of the leading causes of blindness worldwide, with elevated intraocular pressure (IOP) being the most significant modifiable risk factor. The objective of this paper is to assess the effectiveness of Direct Selective Laser Trabeculoplasty (DSLT) in the management of glaucoma and OHT based on analysis of available literature. Topical hypotensive medications are the preferred first-line therapy for both newly diagnosed open angle glaucoma (OAG) and ocular hypertension (OHT) over other treatment modalities for most patients. Medical glaucoma management, despite proven efficacy, is limited by issues of patient adherence, quality-of-life decrease, side effects, and ocular surface disease (OSD). Selective Laser Trabeculoplasty (SLT) has emerged as a safe, effective, and repeatable alternative to medical therapy for patients with open-angle glaucoma (OAG) and ocular hypertension (OHT). Direct Selective Laser Trabeculoplasty (DSLT) is a novel, non-contact laser treatment that delivers energy transsclerally to the trabecular meshwork (TM) without the use of a gonioscope. In recent studies, DSLT has demonstrated comparable efficacy to conventional SLT, including the multicenter randomized GLAUrious trial. It reduces IOP by 18–27%, often enabling reduction in or discontinuation of hypotensive topical medications. The non-contact, automated nature of DSLT simplifies the procedure, enhances patient comfort, and may expand access to laser therapy across diverse clinical settings. In conclusion, DSLT represents an innovative advancement in laser therapy for glaucoma, combining the clinical benefits of SLT with enhanced procedural efficiency and safety. Further long-term studies are needed to validate its durability, but existing evidence supports its use as a first-line or adjunctive treatment for OAG and OHT.

## 1. Introduction

Glaucoma, a chronic multifactorial neuropathy, is characterized by the progressive degeneration of the retinal ganglion cells. It is the most common cause of irreversible blindness, and its global prevalence is estimated to be around 4% in patients between the ages of 40 and 80 years old [1].

While the pathogenesis of glaucoma is complex, intraocular pressure level plays a crucial role in its progression and is the only known modifiable risk factor. Therefore, lowering IOP remains the keystone of glaucoma treatment. In patients with glaucoma, the intraocular pressure can be reduced with regularly applied antiglaucoma topical medications, laser therapy, and surgical interventions [2]. First-line, evidence-based management for glaucoma typically involves topical intraocular pressure (IOP)-lowering medications [3,4]. The significant characteristics of this therapy contribute to its trend as a first-choice treatment. Its effectiveness and safety have been confirmed in many reliable clinical trials. This therapy lowers intraocular pressure to levels that halt the progression of the disease. However, topical glaucoma therapy has several important limitations. The key limitation is the need for patients to self-administer this therapy one or more times daily, often from more than one bottle. Regular use is essential to ensure long-term IOP control. It is well known that nonadherence to topical antiglaucoma medications is a common issue [5]. Irregular use of eye drops has been shown to increase the risk of disease progression [6].

Another limitation of drop therapy is that it has both local and systemic side effects.

Chronic ocular surface disease (OSD) is a frequent disabling challenge among patients with glaucoma. Researchers report that 48–59% of patients with glaucoma experience symptoms of OSD, while 22–78% present clear clinical symptoms [6,7]. Prolonged use of antiglaucoma eye drops, especially those containing benzalkonium chloride (BAK) as a preservative agent, significantly exacerbates OSD [8]. BAK-related ocular toxicity has been demonstrated to reduce the quality of patients’ lives [9].

Chronic therapy leads to numerous adverse effects (irritation, tearing, stinging, conjunctival hyperemia, superficial punctate keratitis, discoloration and edema of the eyelids, changes in iris color, periorbital disorders, and accelerated cataract development), which lead to the irregular use of drugs by patients and consequently result in frequent visits to a specialist [3,10,11].

As the majority of glaucoma medications work by stimulating or inhibiting adrenergic, cholinergic, and prostaglandin receptors, they may also lead to systemic side effects like decreased resting pulse rate, blood pressure, and spirometry values, as well as allergic reactions or exacerbation of comorbidities [12].

Long-term use of topical glaucoma medications is an important risk factor for potential surgical failure due to the activation of conjunctival fibroblasts by medications or preservatives. This effect is thought to be caused by chronic inflammation and scarring in the conjunctiva, which can hinder the formation and function of the filtration bleb after glaucoma surgery [13]. 

This article is a narrative review prepared in accordance with the SANRA guidelines [14]. The aim was to summarize and critically discuss current evidence regarding Direct Selective Laser Trabeculoplasty (DSLT) in the management of glaucoma. A comprehensive search was conducted in PubMed, Embase, and Web of Science databases. The search has covered the period from January 2000 to July 2025. The following keywords and their combinations were used: “*selective laser trabeculoplasty*”, “*SLT*”, “*direct selective laser trabeculoplasty*”, “*DSLT*”, “*normal tension glaucoma*”, “*ocular hypertension*”, “*open-angle glaucoma*”, and “*laser therapy*”. Selection of the papers was conducted following the inclusion (peer-reviewed clinical studies, randomized controlled trials, meta-analyses, systematic and narrative reviews, and experimental studies relevant to SLT and DSLT) and exclusion criteria (non-English publications, case reports, conference abstracts without available full text, and studies not directly addressing glaucoma management). Although formal scoring systems for systematic reviews were not applied during data synthesis, the methodological quality of the included studies was considered. Emphasis was placed on randomized controlled trials, prospective comparative studies, and high-quality reviews to ensure reliable conclusions.

## 2. Selective Laser Trabeculoplasty

Selective Laser Trabeculoplasty (SLT), a common ophthalmic procedure, is a treatment option that overcomes limitations of medical management with no need for regular drug administration and no systemic side effects occurring at the same time, resulting in an effective and safe procedure. The European Glaucoma Society (EGS) recommends SLT as potential initial treatment in patients with mild or moderate open-angle glaucoma (OAG) and ocular hypertension (OHT) [15]. The indications for SLT in OAG include patients with primary, pseudoexfoliation, and pigmentary glaucoma [16]. Some studies have also reported SLT’s effectiveness in lowering IOP, in primary angle-closure glaucoma (PACG) [17,18]. However, the procedure has not been widely adopted in clinical practice due to the complexities of visualizing the trabecular meshwork (TM) in these patients [17,18].

Contraindications for SLT include poor visualization of the TM due to corneal factors and poor patient cooperation. It is not recommended in cases with extensive angle-closure or peripheral anterior synechiae. SLT is contraindicated for treating neovascular glaucoma. It is not advised in cases of congenital glaucoma. Although SLT is not recommended for uveitic glaucoma with active inflammation, quiet eyes with a remote history of uveitis and no recent uveitis episodes may benefit [19].

Selective Laser Trabeculoplasty employs the Q-switched, frequency-doubled, 532-nanometer Nd:YAG laser, which emits a 400 μm diameter treatment spot on the trabecular meshwork (TM) in 3 nanoseconds [20]. The exact mechanism by which SLT lowers IOP remains incompletely characterized, but it is likely multifactorial [21]. Three main theories have been proposed: a mechanical effect, a biochemical effect, and a cellular effect [22,23,24]. At the organ level, SLT’s hypotension effect is based on aqueous humor dynamics change. The treatment increases TM outflow [25,26,27].

Prior studies have shown that trabeculoplasty safely and effectively reduces mean intraocular pressure (IOP) 20–30% (4–6 mmHg) from the baseline [28,29,30,31,32].

Complications are rare and include transient discomfort or mild pain, short-term photophobia, intraocular pressure spikes, inflammatory flare in the anterior chamber, conjunctival hyperemia, corneal scarring or corneal decompensation due to endothelial cell damage, and cystoid macular edema [33,34].

McIlraith et al. reported reductions in IOP in approximately 80% of eyes which underwent SLT, with the remaining 20% having little or no hypotensive effect 12 months after the treatment [32].

A retrospective multicenter study conducted by Khawaja et al. included 831 SLT-treated eyes. They reported significant IOP reductions initially, but 55% of eyes met failure criteria (IOP > 21mmHg or < 20% reduction from the baseline, increase in glaucoma medications, subsequent glaucoma procedure including repeat SLT) within 12 months, increasing to 82% of eyes after 36 months [35].

The Laser in Glaucoma and Ocular Hypertension (LiGHT) Trial is a multicenter randomized controlled trial investigating the outcomes of initial treatment with SLT and initial treatment with IOP-lowering topical medication for treatment-naïve patients with mild to moderate OAG or high-risk OHT. A total of 692 patients were enrolled, assigned to primary SLT or primary medical therapy, and followed for 3 years. Treatment escalations were more common in the medication group compared to the SLT group. Progression rates were higher with the medication group. Of the SLT-treated eyes, 76.6% required only a single SLT during the 3-year observation period, and 78.2% remained medication-free at 3 years. Medically treated eyes were more likely than SLT-treated eyes to have progression [36]. A total of 524 out of the 692 patients completed 6 years of extension in the trial. At 72 months, 69.8% of the SLT group remained at the target IOP or less than the target without the need for medical or surgical treatment. More eyes in the medication group exhibited disease progression [37].

The effectiveness of SLT in normal-tension glaucoma has also been investigated and confirmed [38,39].

Lee et al. presented the outcomes of a prospective cohort study where 41 normal tension glaucoma (NTG) patients treated with antiglaucoma topical medications underwent SLT after the 1-month washout of antiglaucoma medication period. All recruited eyes had open angle on gonioscopy.

The mean pre-study IOP was 14.3 ± 3.4 mmHg, while patients were on 1.5 ± 0.8 antiglaucoma eye drops. The mean baseline IOP after washout was 16.2 ± 2.2 mmHg. 

At 12 months post-SLT, the mean IOP was 12.2 ± 2.2 mmHg while on 1.1 ± 0.9 antiglaucoma eye drops (*p* < 0.001). Authors reported that a single session of SLT for NTG achieved an additional 15% IOP reduction while using 27% less medication at 1 year compared to pre-study levels (*p* < 0.001) [38].

In the multicenter prospective study, Nitta and co-authors investigated and compared the clinical efficacy and safety of first-line and second-line SLT in Japanese patients with NTG. Data for 99 eyes from 99 patients with NTG were included in the analysis: 74 eyes in the first-line SLT group and 25 eyes in the second-line SLT group. The authors reported that the mean IOP of 16.3 ± 2.1 mm Hg before SLT decreased by 17.1 ± 9.5% to 13.4 ± 1.9 mm Hg at 12 months in the first-line group (*p* < 0.001), and the mean IOP of 15.4 ± 1.5 mm Hg before SLT decreased by 12.7 ± 9.7% to 13.2 ± 2.0 mm Hg at 12 months (*p* = 0.005) in the second-line group [39].

Study outcomes support SLT as a first-line treatment for mild to moderate OAG, including the NTG [38,39] and OHT [36,37,40].

## 3. Direct Selective Laser Trabeculoplasty (DSLT)

**Figure 1:**
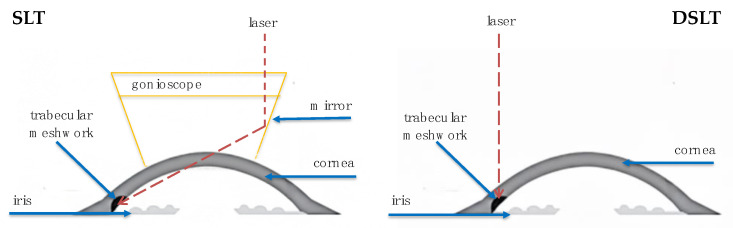
Laser Delivery for SLT and DSLT.

Direct Selective Laser Trabeculoplasty (DSLT) is a modern laser method for lowering intraocular pressure, used in the treatment of open-angle glaucoma and ocular hypertension. Unlike conventional Selective Laser Trabeculoplasty, DSLT does not require the use of a gonioscope. It works through the conjunctiva and sclera, delivering laser pulses directly to the TM. The procedure is completely non-contact, automated, and lasts only a few seconds [41]. The simplicity and speed of the procedure allow it to be performed even by less-experienced operators, which potentially increases its accessibility in everyday clinical practice [42]. Figure 1 depicts laser targeting of the TM through the cornea while SLT and transscleral when DSLT is performed.

The mechanism of action of DSLT is based on the selective effect of laser pulses on pigmented cells located in the trabecular meshwork, resulting in improved aqueous humor outflow and reduced IOP [41].

In the study by Sacks et al., a detailed analysis of light propagation in Direct Selective Laser Trabeculoplasty (DSLT) was conducted to determine the efficacy and safety of this method. DSLT utilizes Nd:YAG laser pulses with a wavelength of 532 nm, which are delivered noninvasively through the conjunctiva and sclera, without the need for a gonioscopy lens or any contact between the device and the patient’s eye. Unlike conventional SLT, in which laser light is applied through a gonioscope and requires precise microscopic alignment, DSLT delivers energy automatically using an external laser system equipped with built-in eye-tracking and a target confirmation system operated by the physician. Using advanced numerical models and experimental measurements, the study evaluated how laser energy penetrates ocular tissues and reaches the TM. It was demonstrated that, in order to achieve the same amount of energy delivered to the trabecular meshwork using DSLT as with SLT, the laser energy in DSLT must be increased by a factor of 2.8 compared to that used in SLT. This is due to the fact that during DSLT, approximately 11% of the energy reaches the TM (compared to approximately 32% in SLT), as determined by simulations and experimental studies [42].

It was also confirmed that, due to scattering and refractive indices within ocular structures, the laser energy is concentrated in the TM area, minimizing exposure to adjacent tissues such as the sclera or ciliary body. The study represents an important link between the physical foundations of the technology and its practical clinical application, supporting the continued development and implementation of DSLT as a modern method for glaucoma treatment [43].

During a single session, the treatment may be applied to one or both eyes of the patient. The procedure can encompass either 180 or 360 degrees of the trabecular meshwork (TM) [40]. There is no single specific protocol for performing Deep Sclerectomy Laser Trabeculoplasty (DLST) procedure. In our clinic, prior to the execution of the DSLT treatment, an alpha agonist glaucoma medication is administered to prevent the post-treatment spike in intraocular pressure (IOP). A few minutes before the initiation of the procedure, a topical anesthetic is applied to enhance patient comfort. An eyelid retractor is inserted immediately prior to the procedure. The treatment can be performed with the patient in either a standing or seated position. Once the patient is correctly positioned, the clinician verifies the location of the limbus as indicated by the device. Subsequently, the DSLT system automatically delivers laser pulses directly through the limbus to the TM, with this process typically lasting between 2 and 3 s [41]. One hour after the procedure, intraocular pressure is measured. We recommend that patients use topical Non-Steroidal Anti-Inflammatory Drug (NSAID) for 7 days after surgery.

### 3.1. Effectiveness of DSLT

In a retrospective study conducted in Leicester, United Kingdom, involving 15 eyes of 10 patients with OAG or OHT, a 25.7% reduction in IOP was demonstrated—from a mean level of 22.7 mmHg to 15.9 mmHg. In 73% of patients, this effect was achieved without the need to modify their existing pharmacological treatment [44]. In a clinical study conducted by Lanza and collaborators, which included 104 eyes of 54 patients with primary open-angle glaucoma (POAG) and primary angle-closure glaucoma (PACG), it was found that the mean intraocular pressure decreased by 3.7 mmHg (POAG: −3.76 ± 2.84 mmHg, PACG: −3.67 ± 2.46 mmHg (*p* < 0.01 vs. baseline), corresponding to a reduction of approximately 18–19%), and the number of pressure-lowering medications used decreased by an average of 0.6 to 0.8 agents.

No serious complications were reported, and perimetrically assessed peripheral visual function remained stable throughout the one-year observation period [45].

The first prospective clinical study on the DSLT technique was conducted by Goldenfeld et al. This nonrandomized study included 15 eyes in patients with OAG, OHT, and pseudoexfoliative glaucoma who were either treated after medication washout or had not yet undergone any therapy. A total of 100 or 120 laser pulses with a wavelength of 532 nm were applied, generated in Q-switched mode, with energy ranging from 0.8 to 1.4 mJ. The procedure lasted from 1.5 to 2.3 s and was performed in a non-contact manner through the limbus, using automatic eye-tracking. The baseline intraocular pressure was on average 26.7 ± 2.3 mmHg. One month after the procedure, it decreased to 21.7 ± 4.2 mmHg, representing a reduction of 18.1%. After three months, the mean pressure was 20.8 ± 2.5 mmHg, a 21.4% decrease, and after six months was 21.5 ± 4.1 mmHg, corresponding to an 18.8% reduction. In the subgroup of six eyes that received the highest pulse energy (1.4 mJ), pressure decreased to 19.3 ± 2.0 mmHg, a reduction of 27.1% (*p* = 0.03). The number of pressure-lowering medications decreased from 1.6 ± 1.0 to 0.4 ± 0.7 agents (also statistically significant, *p* = 0.03). No serious adverse events were reported during the follow-up period. The researchers concluded that energy application at the level of approximately 1.4 mJ is effective and well tolerated, and the procedure has a favorable safety profile [46].

The results of studies conducted by Geffen et al. and Goldenfeld et al. provide important data on the efficacy and safety of DSLT. In Geffen’s study, it was demonstrated that the use of a transscleral pulsed Nd:YAG laser with a wavelength of 532 nm enables effective reduction in intraocular pressure without the need for a gonioscope or direct contact with the eye, which constitutes an advantage over conventional SLT [46]. In the prospective clinical study by Goldenfeld, these observations were confirmed, showing a significant average decrease in IOP and a reduction in the number of antiglaucoma medications in patients with OAG after undergoing DSLT therapy [40].

In 2023, Congdon and colleagues designed the multicenter, randomized, controlled GLAUrious trial aimed at comparing DSLT with conventional SLT in patients over 40 years of age with OAG or OHT, whose baseline intraocular pressure after washout ranged from 22 to 35 mmHg. In the DSLT group, 120 pulses lasting 3 ns, with a diameter of 400 µm and energy ranging from 1.4 to 1.8 mJ, were applied through the limbus over approximately 2 s. In the SLT group, about 100 pulses with the same temporal and spatial parameters but with energy ranging from 0.3 to 2.6 mJ were applied across 360 degrees of the trabecular angle using a gonioscope. The study aimed to enroll 164 participants, with a non-inferiority margin set at 1.95 mmHg [47]. The final results of the GLAUrious study were published in May 2025 by Gazzard and co-authors. A total of 192 patients were randomized, with 99 receiving DSLT and 93 receiving SLT. The procedures were performed at 14 clinical centers in the United Kingdom, Italy, Israel, and Georgia. All patients were treated with 0–3 antiglaucoma medications, and their baseline intraocular pressure after medication washout ranged between 22 and 35 mmHg. In the analysis of 156 patients at 6 months, the mean IOP reduction in the DSLT group was 5.5 ± 0.5 mmHg (20.6%), while in the SLT group it was 6.2 ± 0.5 mmHg (23.6%). The difference was −0.7 mmHg, which fell within the range of statistical insignificance (95% CI: −2.2 to 0.8 mmHg, *p* = 0.09), but it did not formally confirm non-inferiority. After 12 months of follow-up in 161 patients, the average pressure reduction in both groups was identical at 3.2 ± 0.4 mmHg (12.2% for DSLT and 9.4% for SLT, respectively), and the difference between the groups was only 0.01 mmHg, which was statistically significant in favor of non-inferiority (*p* < 0.001). Both procedures demonstrated a similar safety profile, although more minor subconjunctival hemorrhages were reported in the DSLT group, which were clinically insignificant. No serious adverse events occurred, and all observed complications were mild and transient [47,48].

According to data from the manufacturer of the DSLT system, Alcon, after 12 months, 62% of patients in the DSLT group did not require pharmacological treatment, and 81% of patients remained on the same or a reduced number of medications compared to before the procedure [49].

Based on the latest studies by Fossati et al. and Puerto et al., the effectiveness and safety of Direct Selective Laser Trabeculoplasty in the treatment of open-angle glaucoma under routine clinical practice conditions can be confirmed. In the retrospective study by Fossati, patients undergoing DSLT showed a statistically significant reduction in intraocular pressure and the possibility of reducing the number of antiglaucoma medications used. The hypotensive effect was sustained during the 6-month follow-up period, and the procedure itself was well tolerated, with no serious complications reported [44]. Puerto et al., in turn, confirmed the effectiveness of DSLT in short-term follow-up (up to 3 months), demonstrating a clear IOP reduction in the majority of patients, regardless of baseline pressure levels or previous pharmacological treatment. The authors also emphasized the high reproducibility of results and the simplicity of the procedure [50].

DSLT, as a modern, non-contact form of selective trabeculoplasty, demonstrates efficacy comparable to conventional SLT, both in short-term and one-year follow-up [43,46,47]. The reduction in intraocular pressure observed in studies ranged between 18% and 27%, with an additional advantage being a significant decrease in the need for hypotensive medications [46]. DSLT is also characterized by a favorable safety profile and greater patient comfort compared to SLT [43,45,46]. In the case of DSLT, a lower incidence of complications such as inflammatory flare in the anterior chamber and superficial punctate keratitis was recorded, as well as less patient discomfort compared to SLT [46]. Table 1 summarizes the characteristics and outcomes of the studies discussed in this chapter.

### 3.2. Comparison of DSLT and SLT

When comparing DSLT and SLT, several important differences should be noted. DSLT does not require contact with the eye, whereas SLT is a contact procedure that necessitates the use of a gonioscope [42]. DSLT lasts only a few seconds, while SLT can take several or even more than ten minutes [42,43]. The automation of DSLT minimizes the operator’s involvement, in contrast to the manual nature of SLT [46]. Patient’s comfort is significantly higher with DSLT, as it does not require contact with the eye, instillation of anesthetic drops, or use of a gonioscope [43,44]. In clinical studies, the efficacy of DSLT was evaluated as comparable to SLT—both in terms of pressure reduction and the number of medications used [44,45,47]. The safety profile of DSLT is also favorable: no serious adverse events were reported, and minor, transient side effects (such as subconjunctival hemorrhages) occurred only occasionally [44,45]. Despite the very promising results, DSLT still requires further research. Most of the available data come from observations lasting up to 12 months; therefore, long-term analyses covering a period of at least several years are needed [45,46,47]. Additionally, although the GLAUrious trial confirmed the non-inferiority of DSLT compared to SLT, other studies lack comparative data, which makes it difficult to clearly assess the durability of therapeutic effects [47]. In some clinical studies, the number of patients was limited, and differences in the laser parameters used (e.g., number of pulses, energy) indicate the need for standardization [44,45,46]. It is expected that DSLT will be equally effective as SLT in lowering intraocular pressure in all types of glaucoma, including angle-closure glaucoma (ACG). In the case of ACG, direct visualization of the trabecular meshwork (TM) using a gonioscope is difficult, which prevents standard application of SLT. Sacks et al. suggest that DSLT may provide a satisfactory pressure-lowering effect in ACG since, using this method, the TM can be targeted trans-tissue by the laser without the need for angle visualization [43,45] (Table 2).

## 4. Conclusions

In summary, Direct Selective Laser Trabeculoplasty is an innovative, non-contact, fast, and automated method of laser treatment for glaucoma, which in current studies has demonstrated efficacy comparable to conventional SLT, while offering greater patient comfort and a simplified surgical procedure. That is why DSLT may be a promising alternative option for SLT as a first-line or adjunctive treatment for OAG and OHT.

Preliminary clinical data are promising; however, to fully evaluate the DSLT technology, further long-term studies involving larger and more diverse patient populations are necessary.

## Figures and Tables

**Table 1:** Direct Selective Laser Trabeculoplasty (DSLT): Full Comparison of Clinical Study Outcomes.

Study (Ref.)	Design and Setting	Population	N (Eyes/Pts)	Follow-Up	Baseline IOP (Mean ± SD) [95% CI]	End IOP/Key Timepoint (Mean ± SD) [95% CI]	ΔIOP (mmHg, %) [95% CI]	Medications (Change)	Success Definition and Rate	Safety
Geffen et al., 2017 [46]	Prospective RCT, Israel	POAG, PXG	14/14	12 months	20.21 ± 3.19 [18.54–21.88]	6 m 15.50 ± 3.77 [13.53–17.47]; 12 m 16.00 ± 3.31 [14.27–17.73]	6 m −4.7 (−23%); 12 m −4.2 (−21%)	Not detailed	≥15% reduction; 86%	Mild, fewer than SLT
Goldenfeld et al., 2021 [40]	Prospective nonrandomized, Israel	OAG, OHT, PXG	15/15	6 months	26.7 ± 2.3 [25.5–27.9]	6 m 21.5 ± 4.1 [19.4–23.6]	−5.2 (−19%); High-energy subgroup −27%	1.6 ± 1.0 → 0.4 ± 0.7	Not explicit	No serious AE
Lanza et al., 2025 [45]	Prospective cohort, Italy	POAG, PACG	104/54	12 months	Not fully reported	Δ12 m: POAG −3.8 ± 2.8; Δ12 m: PACG −3.7 ± 2.5	As above	Reduced	Not defined	No major AE
Fossati et al., 2025 [44]	Retrospective, UK	OAG, OHT	15/10	4 months	22.7 ± 4.4 [20.5–24.9]	4 m 18.7 ± 4.2 [16.6–20.8]	−4.0 (−17.6%)	Not detailed	73% success	No major AE
Puerto et al., 2025 [50] Group 1	Retrospective, Spain	First-line DSLT	20/—	6 months	26.50 ± 2.70 [25.32–27.68]	6 m 20.35 ± 3.37 [18.87–21.83]	−6.1 (−23%)	All drop-free	≥20% reduction;	No AE
Puerto et al., 2025 [50] Group 2	Retrospective, Spain	Burden reduction	28/—	6 months	15.36 ± 4.34 [13.75–16.97]	6 m 14.75 ± 4.15 [13.21–16.29]	−0.6 (−4%)	Stable	Not defined	No AE
GLAUrious Trial, 2025 [47]	Multicenter RCT, DSLT vs. SLT	OAG, OHT	192/—	6 and 12 months	Not detailed (screening baseline)	6 m DSLT −5.5 ± 0.5 [−6.48–4.52], SLT −6.2 ± 0.5 [−7.18–5.22]; 12m both −3.2 ± 0.4 [−3.98–2.42]	6 m diff −0.7 (95%CI −2.2 to 0.8); 12 m diff 0.01 (95%CI −1.1 to 1.1)	62% drop-free at 12 m	Similar to SLT; mild hemorrhages more frequent DSLT	Non-inferiority confirmed

**Table 2:** Comparison of SLT and DSLT.

Feature	SLT (Selective Laser Trabeculoplasty)	DSLT (Direct Selective Laser Trabeculoplasty)
Type of procedure	Contact procedure (requires gonioscope and slit-lamp microscope)	Non-contact (laser applied through conjunctiva and sclera)
Duration	Several to over ten minutes	A few seconds
Operator involvement	Manual positioning and pulse delivery by the physician	Automated procedure with eye-tracking and target confirmation system
Patient comfort	Requires topical anesthesia and gonioscope contact with the eye	Higher comfort—no contact, no anesthetic drops, no gonioscope needed
Mechanism of action	532 nm Nd:YAG laser pulses → selective effect on pigmented trabecular meshwork (TM) cells → improved aqueous humor outflow	Similar mechanism—532 nm Nd:YAG laser energy delivered through conjunctiva and sclera; requires ~2.8 × higher energy compared to SLT due to tissue transmission losses
Efficacy (IOP reduction)	20–30% (average 4–6 mmHg)	18–27% (average 3–6 mmHg), comparable to SLT (GLAUrious trial)
Impact on medical therapy	Allows reduction in or discontinuation of topical medication (e.g., LiGHT trial: ~70% drop-free after 6 years)	Also enables reduction in or discontinuation of medication (e.g., 62% medication-free after 12 months per Alcon data)
Safety	Good, possible transient inflammation, discomfort, subconjunctival hemorrhage	Very good, no serious adverse events; rare, mild subconjunctival hemorrhages reported
Repeatability	Repeatable, although efficacy may decline with repeated sessions	Repeatable, but long-term evidence is still limited
Limitations	Requires gonioscopy—difficult/impossible in angle-closure glaucoma	Can be applied in angle-closure glaucoma (no need for angle visualization)
Clinical evidence	Extensive data, including large long-term trials (LiGHT trial—6 years)	Growing evidence, including GLAUrious trial (2025, 12-month results); long-term studies still needed

## Data Availability

The data that support the article content were taken from the previously published papers. All the source texts are listed in References.
